# Infrared Spectroscopy of Pentagon-Containing PAHs: Indenyl and Fluorenyl Anions and Indenyl Cation

**DOI:** 10.1021/acs.jpclett.5c00570

**Published:** 2025-04-11

**Authors:** Gabi Wenzel, Miguel Jiménez-Redondo, Milan Ončák, Brett A. McGuire, Sandra Brünken, Paola Caselli, Pavol Jusko

**Affiliations:** † Department of Chemistry, Massachusetts Institute of Technology, 77 Massachusetts Avenue, Cambridge, Massachusetts 02139, United States; ‡ Center for Astrophysics/Harvard & Smithsonian, 60 Garden Street, Cambridge, Massachusetts 02138, United States; ¶ Max Planck Institute for Extraterrestrial Physics, Giessenbachstrasse 1, 85748 Garching, Germany; § Institute for Ion and Applied Physics, University of Innsbruck, Technikerstraße 25, Innsbruck 6020, Austria; ∥ National Radio Astronomy Observatory, 520 Edgemont Road, Charlottesville, Virginia 22903, United States; ⊥ Radboud University, Institute for Molecules and Materials, FELIX Laboratory, Toernooiveld 7, 6525 ED Nijmegen, The Netherlands

## Abstract

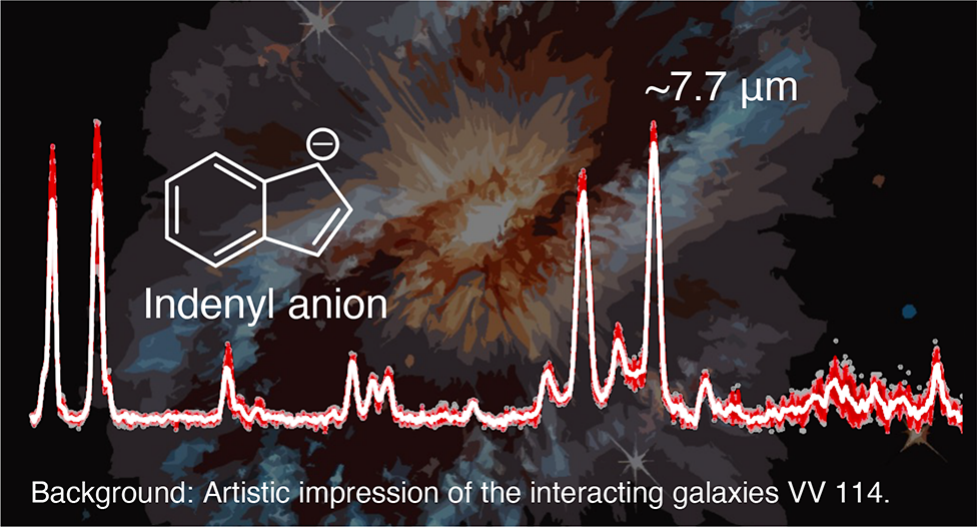

Polycyclic aromatic hydrocarbon (PAH) ions are crucial intermediates in interstellar chemistry and may play a key role in the infrared emission features observed in space. Here, we investigate the infrared spectra of the indenyl (C_9_H_7_^–^) and fluorenyl (C_13_H_9_^–^) anions and the indenyl cation (C_9_H_7_^+^) using infrared predissociation (IRPD) spectroscopy. The experiments were performed in a cryogenic 22 pole ion trap at the FELion beamline of the tunable free electron laser FELIX. Spectral analysis of the two anionic PAHs, in combination with density functional theory (DFT) computations, revealed key vibrational modes near 1300 cm^–1^, making these ions potential carriers of the 7.7 μm PAH emission band seen in many astronomical objects. The feature-rich spectrum of cationic indenyls could not be entirely explained by modeling through time-independent anharmonic DFT calculations. Although a better match has been achieved through molecular dynamics simulations, we cannot completely rule out the presence of multiple cationic isomers of the H-loss fragments of indene in the experiments.

The physics and chemistry of the interstellar medium (ISM) are governed by molecular processes involving organic matter that are intimately linked to the different stages of the stellar evolution cycle. Approximately 10–20% of the cosmic carbon content is locked up in polycyclic aromatic hydrocarbons (PAHs),^1^ and their presence in the ISM has long been proposed due to the observations of the aromatic infrared bands (AIBs) in many astronomical objects.^2^ In these regions, isolated PAHs are photoexcited by ultraviolet (UV) photons. One of their major relaxation channels is vibrational relaxation resulting in the emission of infrared (IR) photons that give rise to the AIBs.^3^ AIB spectra have been used to study the bulk composition of astrophysical PAHs and their structure, functionalization, and charge state.^4^ The identification of a specific PAH from AIB spectra, however, remains elusive due to the broad emission features resulting from overlapping bands of many different PAHs. The cold and dark Taurus Molecular Cloud 1 (TMC-1; *T* ≈ 10 K) has proven to be a molecule-rich environment, and to date, nine PAHs have been unambiguously detected in radio-astronomical observations of this source. A majority of these consist of nitrile-functionalized PAHs due to their increased permanent electric dipole moments, namely, 2-cyanoindene,^5^ 1- and 2-cyanonaphthalene,^6^ 1- and 5-cyanoacenaphthylene,^7^ and 1-, 2-, and 4-cyanopyrene.^8,9^ The only pure (unsubstituted) PAH indene was detected both in GOTHAM observations with the 100 m Robert C. Byrd Green Bank Telescope^10^ and in QUIJOTE observations using the Yebes 40 m radio telescope.^11^ Two possible gas-phase formation pathways, one of which occurs at low temperatures, have only recently been uncovered.^12,13^ Many of the detected PAHs contain pentagonal structures, all based on cyclopentadiene,^11^ including two isomers of cyanocyclopentadiene^14,15^ together with two ethylnylated cyclopentadiene isomers,^11^ and the aforementioned indenes and cyanoacenaphthylenes. This is remarkable because these mark the first radio-astronomical detections of molecular species with pentagonal structures. On the other hand, the fullerene family of molecules (C_60_, C_60_^+^, and C_70_), the largest molecules detected so far in space, was found via optical and infrared measurements and contains (exclusively) pentagonal and hexagonal carbon rings.^16,17^ The fully dehydrogenated versions of indene and fluorene, and their charged variants, are direct building blocks of fullerenes.

Of the more than 300 molecules detected in the ISM to date, only eight are anions, and these have only been observed in the past 20 years.^18^ None of them are cyclic species; however, successful detections demonstrate that large hydrocarbon anions containing as many as 10 carbon atoms are present,^19−24^ and models predict that PAH anions are the dominant carriers of negative charge in cold and dense molecular clouds like TMC-1,^25^ where both the anions and PAHs mentioned above were detected. It is worth noting that the distinction between “molecules” and “grains” is generally a matter of convention, and grains as small as 3 Å (approximately the size of an indenyl molecule) are considered in studies of collisional charging of interstellar grains.^26^ In general, the charge states of PAHs in hotter and UV-irradiated environments such as photodissociation regions (PDRs) have been constrained to their neutral or cationic forms while their electron affinities (*E*_ea_) show that PAHs, and in particular dehydrogenated radical PAHs, are viable candidates for electron attachment.^27−30^ This ability is enhanced when a pentagonal ring is present in the molecule,^31^ making the study of these anions crucial to expanding our understanding of the physicochemical processes at play in the ISM.

Here, we aim to investigate the pentagon-containing, planar PAH species indenyl (C_9_H_7_, *m*/*z* 115) and its related extension fluorenyl (C_13_H_9_, *m*/*z* 165) in their anionic and cationic states using infrared predissociation (IRPD) spectroscopy. Their molecular structures are depicted in the insets of Figure 1. The electron affinities for the indenyl and fluorenyl radicals have been previously determined to be 42.7 ± 0.3 kcal/mol (1.852 ± 0.013 eV) and 43.1 ± 0.3 kcal/mol (1.869 ± 0.013 eV), respectively,^32^ making them prone to electron attachment.

**Figure 1:**
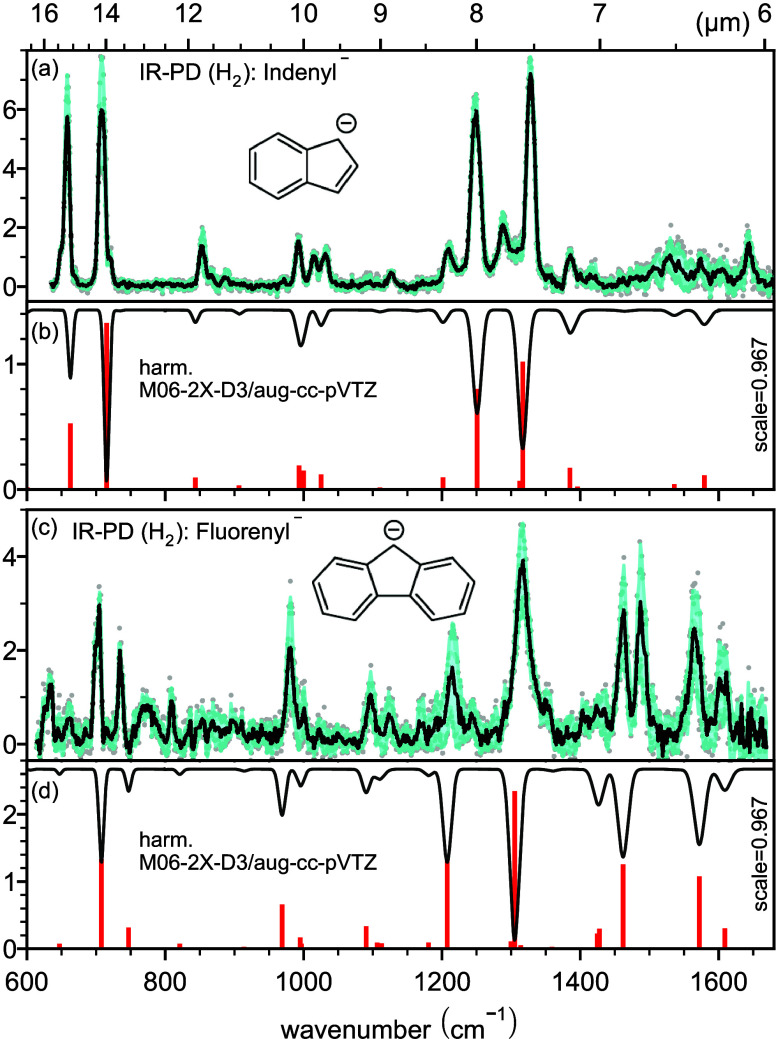
IRPD spectra (black) of the (a) indenyl and (c) fluorenyl anions tagged with H_2_ resulting from averaging all measurements for each wavenumber step (gray dots) with the error envelope (cyan shading). The corresponding harmonic frequency stick spectra computed at the M06-2X-D3/aug-cc-pVTZ level of theory are colored red with inverse intensity (panels b and d). A scaling factor of 0.967 was applied. The inverted traces represent the computed spectra convolved with Gaussians with a bandwidth of 0.5% of the corresponding wavenumber representative for the FELIX-2 laser line profile.

Indenyl and fluorenyl anions were previously spectroscopically examined by means of photoelectron velocity-map imaging spectroscopy;^33^ approximately 20 and 30 well-resolved vibronic transitions were identified from the anion and the radical neutral, respectively. An attempt to assign some of these features to theoretical spectra computed by density functional theory (DFT) delivered limited success, and no information about the vibrational modes of the anions was deduced. The anionic indenyl photodetachment spectrum was later revisited by Kumar et al.^34^ Using *ab initio* (MP2) calculations, they assigned the fundamental, combination, and overtone bands of the neutral radicals and calculated values for the anion vibrational modes. Thus, to date, there are no experimental data on the vibrational spectra of the indenyl and fluorenyl anions and only limited direct vibrational gas-phase spectroscopy of radical [PAH]^−^ and deprotonated [PAH–H]^−^ anions in general. Gao et al.^35^ studied 2-naphthyl, 9-anthracenyl, and 1-pyrenyl, using IR multiphoton electron detachment inside a Fourier transform ion-cyclotron resonance (FT-ICR) trap, and Salzmann et al.^36^ recorded the CH stretching region of the Ar-tagged pyrene anion formed in a supersonic expansion.

It is interesting to note that, whereas the indenyl and fluorenyl anions are aromatic species, the cations do not fulfill Hückel’s rule for aromaticity because they contain 4*n* π-electrons, 8 and 12, respectively, and therefore are considered anti-aromatic. This is also supported by our computational nucleus-independent chemical shift (NICS) analysis (see section S4 of the Supporting Information (SI)). Nevertheless, the stability of the fluorenyl cation has been demonstrated as it was identified as a common fragment in IR multiple-photon dissociation (IRMPD) spectra when dissociating 9,10-dihydrophenanthrene, 9,10-dihydroanthrancene, and fluorene cations in ion trap experiments.^37^ The electronic spectrum of the indenyl cation embedded in a solid Ne matrix revealed only four broad bands positioned up to 2000 cm^–1^ with tentative assignments using DFT calculations.^38^ The fluorenyl cation has also been isolated in cold matrices. Its IR absorption spectrum has been recorded in an amorphous water/ice matrix below 30 K,^39^ while the electronic spectrum was measured in a Ne matrix at 6 K, together with those of other isotopologues of C_13_H_9_^+^.^40^ One of the most recent studies revealed a less perturbed IR spectrum of the ultracold fluorenyl cation and its two functionalized siblings in a He nanodroplet experiment (≈10^5^ He atoms at 0.4 K) using a cryogenic (90 K) hexapole ion trap accompanied by matching DFT calculations.^41^ Other recent studies concentrated on CN-functionalized hydrocarbons, namely, the 2-cyanoindene^42^ and cyanocyclopentadiene cations.^43^

All of the species mentioned here contain the pentagonal C_5_ ring as a not fully saturated hydrocarbon, such as cyclopentadiene. From a molecular physics perspective, the concept of (anti)aromaticity is pivotal in understanding the structure and stability of such polycyclic hydrocarbons, and introducing a charge into pentagon-containing PAHs will make a system (anti)aromatic with unique electronic properties. As for the above-mentioned cyclopentadiene, in its anionic charge state, it is considered to be aromatic, while its cationic counterpart is anti-aromatic.^44,45^ We point out that two other cations with 4*n* + 2 π-electrons have recently been studied with IR and UV predissociation experiments, and their vibrational and electronic spectra were recorded: *n* = 0 for c-C_3_H_3_^+^,^46^ and *n* = 1 for c-C_7_H_7_^+^.^47,48^

Here, we use IRPD spectroscopy with a molecular hydrogen (H_2_) tag for the ions of interest probed in the cryogenic 22 pole ion trap tandem mass spectrometer, FELion,^49^ coupled to the widely tunable free electron lasers at the FELIX Laboratory (see Experimental Methods for details).^50^

The recorded IRPD spectra of the indenyl and fluoroenyl anions tagged with H_2_ are presented in panels a and c, respectively, of Figure 1. We compare them to predicted spectra of these anions determined using harmonic theoretical calculations at the M06-2X-D3/aug-cc-pVTZ level of theory^51,52^ that have been scaled with an empirical factor of 0.967 (factor for this method/basis set according to CCCBDB (https://cccbdb.nist.gov/vsfx.asp)) to obtain a good match to the experimental spectra; these calculated spectra are depicted in panels b and d of Figure 1. We can assign several fundamental bands by comparing their band centers to out-of-plane CH (γ_CH_), in-plane CH bending (δ_CH_), and CC stretching (ν_CC_) modes, or modes with mixed character of these as listed in Table S3.1.

For the indenyl anion, the four strongest modes are at 658, 709, 1249, and 1328 cm^–1^ which we can assign to γ_CH_ (658 and 709 cm^–1^) and mixed character ν_CC_ and δ_CH_ (1249 and 1328 cm^–1^) modes. Most of the weaker features in the IRPD spectrum of C_9_H_7_^–^ are accounted for in our theoretical harmonic IR frequencies; however, features at 1015 and 1290 cm^–1^, and those above 1400 cm^–1^, do not have clear counterparts in the theoretically computed harmonic IR spectrum of the indenyl anion and therefore cannot be assigned. They might belong to combination bands of the indenyl anion or are shifted from the calculated values due to anharmonic shifts that are unaccounted for. Although we cannot completely rule out the presence of another C_9_H_7_^–^ isomer in low abundance and/or concentration, none of the calculated spectra of selected isomers indicate that this would be the case (see Figure S5.1).

The IRPD spectrum of C_13_H_9_^–^ can be assigned to the fluorenyl anion, and most of the observed IRPD bands have a corresponding harmonic frequency counterpart. The two strongest modes of the fluorenyl anion at 703 and 1316 cm^–1^ belong to the out-of-plane CH (γ_CH_) mode and a mode with a mixed character of the CC stretch and in-plane CH bending motions (ν_CC_ and δ_CH_), respectively. In the range of 1400–1500 cm^–1^, the assignment is not so clear. There are two strong bands in the IRPD spectrum of C_13_H_9_^–^ located at 1461 and 1487 cm^–1^, while a much weaker feature appears at 1421 cm^–1^. The latter might be assigned to the ν_CC_ + δ_CH_ mode of the fluorenyl anion at 1424 cm^–1^ and matching the 1461 cm^–1^ mode with the 1462 cm^–1^ mode. However, this would leave the second strongest IRPD feature at 1487 cm^–1^ unassigned. We therefore propose also an alternative assignment, which is listed in parentheses in Table S3.1, considering the feature at 1421 cm^–1^ to be in the noise of the experiment. In any case, the origin and assignment of these features are not completely clear since we could also expect the overtone of the strong γ_CH_ out-of-plane CH bending mode in this region.

Notably, one of the strongest features in the IRPD spectra of both anionic species arises between 1250 and 1330 cm^–1^ (8.0 to 7.5 μm) and corresponds to CC stretching modes. As such, it is one of the most prominent PAH emission features centered around 7.7 μm, which plays a significant role in tracing PAH populations in the ISM that are influenced by the local radiation field and environmental conditions.^53,54^ Just recently, detection of this band in unprecedented spatial resolution using the James Webb Space Telescope (JWST) uncovered star formation processes in luminous infrared galaxy VV 114,^55^ consisting of the ongoing interaction or merger of two galaxies. Our measurements show that the indenyl and fluorenyl anions could contribute to this prominent PAH feature in regions with strong UV radiation fields.

For the indenyl cation, the spectral assignment is somewhat less conclusive based on scaled harmonic calculations alone, as shown in Figure S5.2b. In particular, the predicted strong mode at 1150 cm^–1^ (mixed character CH and CC in-plane bending) is not present in the experimental spectrum. Other isomeric structures explored theoretically at the same level of theory do not provide a conclusive match either (see Figure S5.2c–h). However, similar to our previous studies on the cationic H-loss fragments of 2-methylanthracene^56^ and aniline^43^ or the cationic C_2_H_2_-loss fragment of anthracene and phenanthrene,^57^ we cannot exclude the presence of several isomers in the experimental spectrum. We thus performed additional anharmonic calculations as well as molecular dynamics simulations, which are shown together with the experimental IRPD spectrum in Figure 2. As one can see, the molecular dynamics calculations capture most of the observed features, although the calculated and measured intensities are mismatched, in particular, below 1000 cm^–1^. Remarkably, the strong 1150 cm^–1^ feature discussed above is weaker in intensity and now matches the observed experimental features, whereas the experimental band at 1100 cm^–1^ is now either shifted or entirely absent. Similarly, a large deviation is seen around 1400–1500 cm^–1^. The anharmonic DFT calculations predict the low-frequency modes better as well as the two experimental modes mentioned above but fail to reproduce the experimental spectrum in other frequency regions. Within the time-independent calculations, most intense absorptions below 800 cm^–1^ correspond to out-of-plane CH bending (γ_CH_), followed by in-plane CH bending (δ_CH_) up to 1500 cm^–1^, followed by CC stretching (ν_CC_) above this wavenumber.

**Figure 2:**
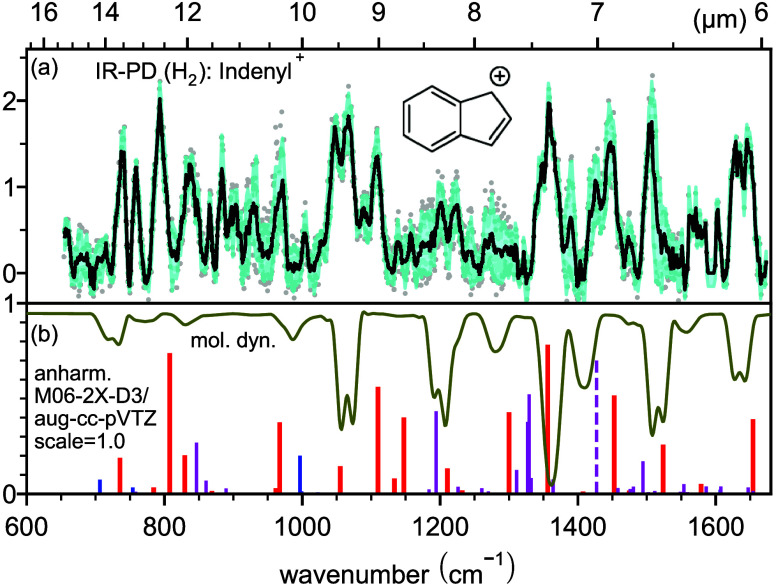
(a) IRPD spectrum (black) of the indenyl cation tagged with H_2_ resulting from averaging all measurements for each wavenumber step (gray dots) with the error envelope (cyan shading). (b) Corresponding frequency stick spectrum computed at the anharmonic M06-2X-D3/aug-cc-pVTZ level of theory: red for fundamental bands, blue for first overtones, and magenta for combination bands. The theoretically calculated intensity is in 100 kJ mol^–1^ (note the unreliability for overtones/combination modes (dashed sticks); see the text). No frequency scaling factor was applied. The inverted trace (olive) represents molecular dynamics simulation at the PBE-D3/DZVP level of theory.

In conclusion, we recorded the IRPD spectra of three pentagon-containing PAH ions, namely, C_9_H_7_^–^, C_13_H_9_^–^, and C_9_H_7_^+^. In the case of the anionic species, we could assign our IRPD spectra by comparison to DFT-calculated harmonic IR modes of indenyl and fluorenyl anions, respectively. To the best of our knowledge, these are the first experimental gas-phase mid-IR spectra of these PAH anions, adding to the sparse existing experimental data on deprotonated PAH anions. We attempted to assign the IRPD spectrum of the cationic H-loss fragment of the indene, C_9_H_7_^+^, to the indenyl cation. However, the IRPD spectrum was convoluted in a way that neither harmonic nor anharmonic DFT calculations at the M06-2X-D3/aug-cc-pVTZ level of theory could fully account for the observed IR features. We note that due to the perturbation treatment, the intensities of the overtones might not be correctly predicted. Molecular dynamics simulations were employed to compute a theoretical IR spectrum of the indenyl cation, which provides a more favorable match. The vibrational spectrum of the indenyl cation seems thus to be influenced by dynamic effects, making it unsuitable for static, time-independent DFT calculations, a method often used to interpret astronomical observations. The presence of other cationic isomers of C_9_H_7_^+^ cannot, however, be excluded. With the identification of the indenyl and fluorenyl anions as contributors to the 7.7 μm PAH emission band, these experiments pave the way for further benchmarking time-independent DFT calculations that can be used to analyze AIB spectra of PDRs and associated astronomical objects with high UV radiation fields, such as Titan’s upper atmosphere in which negatively charged PAHs are also hypothesized to be present.^58−61^ Especially with the large amount of new JWST data, further studies of PAH anions become necessary. In this context, we highlight the recent advancements of the implantation of anions into He nanodroplet matrices, in which even closed-shell PAHs with low electron affinities (*E*_ea_ ≪ 0.5 eV) can form stable anions upon attachment of thermal electrons.^62,63^ This might provide a viable technique for future spectroscopic studies.

## Experimental Methods

The spectroscopic experiments were performed in the cryogenic radio frequency 22 pole ion trap^64,65^ located at the FELion beamline^49^ at the Free Electron Lasers for Infrared eXperiment (FELIX) Laboratory.^50^ Indene (C_9_H_8_) and fluorene (C_13_H_10_) neutral samples (Sigma-Aldrich, purities of ≥99% and 98%, respectively) were used without further purification. These precursors were vaporized by careful heating of the sample reservoir and directly fed into a storage ion source.^64^ The indenyl and fluorenyl ions were produced by electron bombardment in dissociative ionization (indenyl cation) and dissociative electron attachment (anions), delivering positively or negatively charged H-loss fragments of the precursor species. This procedure was first optimized in a similar 22 pole ion trap setup, Cold CAS Ion Trap (CCIT),^66^ where an optimum electron energy, *E*_e^–^_, of approximately 50 eV for anion production has been found. The produced ions were subsequently mass selected using a quadrupole mass filter and injected into the cryogenically cooled 22 pole ion trap held at approximately 10 K to avoid H_2_ freeze-out. Large quantities of trapping He gas were pulsed into the trap at the same time as ion injection in order (a) to trap the ions and (b) to remove their internal and kinetic energy, i.e., cool the ions to temperatures close to that of the trap wall. After a set storage time, the ions were extracted and mass selected in a second quadrupole mass filter prior to detection using an MCP counting detector. This experimental sequence, describing one cycle, was repeated over a typical period of 3 s. In order to switch between the cation and anion operation of the setup, only the relevant electrical potentials had to be adjusted.

In IRPD spectroscopy, the action scheme of dissociating a previously formed ion–tag complex upon resonant IR photon excitation is applied. Here, a 3:1 He/H_2_ gas mixture was used as a trapping gas in order to promote ternary H_2_ attachments during the injection period. Therefore, the ion–tag complexes are efficiently produced only during the initial high-number density injection period (approximately the first 100 ms; total number density of >10^15^ cm^–3^), and no other reaction influences the number of ions inside the trap during the remaining storage time. We found that the He and Ne ternary sticking reaction rates were too low to attach these noble gases to any of the studied charged species in quantities sufficient for a reliable spectroscopic study. The widely tunable mid-IR light of the free electron laser, FELIX-2 (www.ru.nl/felix/), operated at a macropulse repetition rate of 10 Hz, delivered on the order of 10 mJ into the ion trap. The spectra, i.e., the number of remaining ion–tag complexes at the end of the storage time and after laser irradiation (typically 2.6 s) inside the ion trap as a function of the laser wavenumber, are presented in Figures 1 and 2. Intensities were baseline corrected to take into account any background processes and normalized to the number of ions in the trap, the number of laser pulses, and the number of photons.^46,49^ The resulting signal is plotted as a positive quantity, and the spectral resolution is only limited by the laser bandwidth. The laser wavelength was calibrated using a grating spectrometer, resulting in typical calibration uncertainties of 1–3 cm^–1^.

## Computational Methods

Infrared spectra of the ions were calculated through both time-independent and time-dependent approaches. As for time-independent approaches, we used several density functionals (M06-2X, ωB97XD, and B3LYP) along with the aug-cc-pVTZ basis set (see the SI for benchmarking). Here, frequencies were calculated using both harmonic approximation and anharmonic calculations within second-order perturbation theory. In time-dependent calculations, we employed molecular dynamics at a temperature of 100 K at the PBE/DZVP level with a time step of 0.5 fs and a total running time of at least 100 ps. Along the trajectory, Wannier centers were calculated every five steps and subsequently used to produce the vibrational spectra. Aromaticity was analyzed using the NICS.^67^ Time-independent calculations were performed in Gaussian,^68^ and molecular dynamics in CP2K;^69^ analysis of the molecular dynamics was performed in TRAVIS.^70,71^

## Data Availability

The data set associated with this work is available under 10.5281/zenodo.10572237.

## Supporting Information Available

Calculated structural parameters, harmonic/anharmonic frequencies, NICS, and harmonic frequencies for isomers of C_9_H_7_^+^ and C_9_H_7_^–^ (PDF)
Transparent Peer Review report available (PDF)
