# Supplementary material for: Infrared Spectroscopy of Pentagon-Containing PAHs: Indenyl and Fluorenyl Anions and Indenyl Cation
Source: J Phys Chem Lett. 2025 Apr 11;16(16):3938–44. doi: 10.1021/acs.jpclett.5c00570 (PMC12035856; doi:10.1021/acs.jpclett.5c00570)
Supplement: Supplementary file 1 — jz5c00570_si_001.pdf [file jz5c00570_si_001.pdf]

# Supporting Information: Infrared Spectroscopy of Pentagon-Containing PAHs: Indenyl and Fluorenyl Anions and Indenyl Cation

Gabi Wenzel,[†,‡,#] Miguel Jiménez-Redondo,[¶,#] Milan Ončák,[§] Brett A. McGuire,[†,‖] Sandra Brünken,[⊥] Paola Caselli,[¶] and Pavol Jusko[*,¶]

†*Department of Chemistry, Massachusetts Institute of Technology, 77 Massachusetts Ave, Cambridge, MA 02139, USA*

‡*Center for Astrophysics | Harvard & Smithsonian, 60 Garden St, Cambridge, MA 02138, USA.*

¶*Max Planck Institute for Extraterrestrial Physics, Giessenbachstrasse 1, 85748 Garching, Germany*

§*Institute for Ion and Applied Physics, University of Innsbruck, Technikerstraße 25, Innsbruck 6020, Austria*

‖*National Radio Astronomy Observatory, 520 Edgemont Rd, Charlottesville, VA 22903, USA*

⊥*Radboud University, Institute for Molecules and Materials, FELIX Laboratory, Toernooiveld 7, 6525ED Nijmegen, The Netherlands*

#*Contributed equally to this work*

E-mail: pjusko@mpe.mpg.de

# Contents

**Data Availability** **SI-2**

**S1 Calculated Structural Parameters** **SI-2**

**S2 Calculated Frequencies** **SI-3**

S2.1 Harmonic (anions: $C_9H_7^-$, $C_{13}H_9^-$) . . . . . . . . . . . . . . . . . . . . . SI-3

S2.2 Anharmonic (indenyl cation) . . . . . . . . . . . . . . . . . . . . . . . . . . . . SI-5

**S3 Band Assignments of IRPD Spectra** **SI-7**

**S4 Nucleus-Independent Chemical Shifts (NICS)** **SI-8**

**S5 Possibility of Isomers** **SI-9**

S5.1 Indenyl Anion . . . . . . . . . . . . . . . . . . . . . . . . . . . . . . . . . . . SI-9

S5.2 Indenyl Cation . . . . . . . . . . . . . . . . . . . . . . . . . . . . . . . . . . SI-10

# Data Availability

All the data presented in this SI, and data at other levels of theory, are also available in machine readable format (text files, scripts, etc.) in the data set associated with this publication at https://doi.org/10.5281/zenodo.10572237.

# S1 Calculated Structural Parameters

File S1.1: Structure of the indenyl anion $C_9H_7^-$ (M06-2X-D3/aug-cc-pVTZ).

```
16

C −0.000000 0.725195 0.283249
C −0.000000 1.408700 −0.945970
C −0.000000 0.707035 −2.134016
C −0.000000 −0.707035 −2.134016
C −0.000000 −1.408700 −0.945970
C −0.000000 −0.725195 0.283249
H −0.000000 2.494607 −0.959938
H −0.000000 1.240490 −3.077607
```

```
H −0.000000 −1.240490 −3.077607
H −0.000000 −2.494607 −0.959938
C 0.000000 −1.151934 1.630964
C 0.000000 1.151934 1.630964
C 0.000000 −0.000000 2.432597
H 0.000000 −2.175942 1.976472
H 0.000000 −0.000000 3.515835
H 0.000000 2.175942 1.976472
```

File S1.2: Structure of the fluorenyl anion $C_{13}H_9^-$ (M06-2X-D3/aug-cc-pVTZ).

```
22

C 0.000000 1.137455 0.930059
C 0.000000 0.000000 1.763367
C −0.000000 −1.137455 0.930059
C 0.000000 −0.716308 −0.449499
C 0.000000 0.716308 −0.449499
C 0.000000 −2.524187 1.202759
C 0.000000 −3.431814 0.166609
C 0.000000 −3.010605 −1.179858
C 0.000000 −1.659053 −1.477124
H 0.000000 −1.331898 −2.512031
H 0.000000 −3.745718 −1.974579
H 0.000000 −4.493324 0.387630
H 0.000000 −2.874086 2.229467
H 0.000000 0.000000 2.843462
C 0.000000 2.524187 1.202759
C 0.000000 1.659053 −1.477124
C 0.000000 3.431814 0.166609
C 0.000000 3.010605 −1.179858
H 0.000000 2.874086 2.229467
H 0.000000 4.493324 0.387630
H 0.000000 3.745718 −1.974579
H 0.000000 1.331898 −2.512031
```

File S1.3: Structure of the indenyl cation $C_9H_7^+$ (M06-2X-D3/aug-cc-pVTZ).

```
16

C 0.000000 0.707720 0.244926
C −0.000000 1.415942 −0.918449
C −0.000000 0.680915 −2.150352
C −0.000000 −0.680915 −2.150352
C −0.000000 −1.415942 −0.918449
C −0.000000 −0.707720 0.244926
H −0.000000 2.498016 −0.931592
H −0.000000 1.223832 −3.084475
H −0.000000 −1.223832 −3.084475
H −0.000000 −2.498016 −0.931592
C 0.000000 −1.122247 1.638302
C 0.000000 1.122247 1.638302
C 0.000000 0.000000 2.460808
H 0.000000 −2.149965 1.978229
H 0.000000 0.000000 3.537703
H 0.000000 2.149965 1.978229
```

# S2 Calculated Frequencies

## S2.1 Harmonic (anions: $C_9H_7^-$, $C_{13}H_9^-$)

File S2.1: Unscaled harmonic frequencies of indenyl anion $C_9H_7^-$.

| B3LYP–D3 | | M062X–D3 | | wB97XD | |
|---|---|---|---|---|---|
| 212.2123 | 6.5823 | 214.6616 | 6.8449 | 215.0503 | 6.7792 |
| 236.7907 | 0.0 | 237.6693 | 0.0 | 239.7029 | 0.0 |
| 394.2234 | 6.2406 | 388.576 | 5.6973 | 396.295 | 6.3187 |
| 432.8986 | 14.6308 | 435.3034 | 15.1582 | 438.631 | 15.6356 |
| 552.0989 | 1.1504 | 551.0456 | 0.9987 | 556.4555 | 1.289 |
| 591.7472 | 0.0 | 597.6591 | 0.0 | 601.528 | 0.0 |
| 597.3147 | 0.3215 | 597.6955 | 0.2301 | 602.9747 | 0.2889 |
| 611.9062 | 1.1003 | 620.9415 | 1.893 | 622.2485 | 0.7617 |
| 652.1181 | 0.0 | 674.1526 | 0.0 | 665.9449 | 0.0 |
| 669.2819 | 60.6319 | 685.431 | 52.7846 | 681.128 | 68.5483 |
| 724.6505 | 128.0575 | 739.7081 | 132.7945 | 740.2351 | 128.8993 |
| 752.8541 | 0.0819 | 758.2905 | 0.8614 | 760.8808 | 0.5331 |
| 763.8122 | 0.0 | 773.2256 | 0.0 | 776.868 | 0.0 |
| 827.4031 | 0.0 | 843.0231 | 0.0 | 844.5201 | 0.0 |
| 849.5094 | 0.0156 | 872.2323 | 9.5481 | 873.4236 | 0.0836 |
| 868.1014 | 9.5474 | 876.1445 | 0.0405 | 876.5212 | 10.2376 |
| 881.5329 | 0.9565 | 879.7755 | 0.6051 | 888.3038 | 0.6655 |
| 912.9579 | 3.6503 | 937.5131 | 3.2771 | 936.3311 | 3.2314 |
| 935.6839 | 0.0 | 958.5268 | 0.0 | 958.8245 | 0.0 |
| 1014.5349 | 26.0332 | 1027.3616 | 19.1549 | 1028.5353 | 22.3963 |
| 1037.4635 | 15.5823 | 1034.1088 | 15.0703 | 1041.345 | 17.5918 |
| 1054.2804 | 13.1909 | 1060.2445 | 12.0903 | 1066.2963 | 13.6094 |
| 1119.5459 | 0.0648 | 1124.0123 | 0.1417 | 1130.6177 | 0.1004 |
| 1160.9627 | 4.3941 | 1148.2162 | 1.5873 | 1160.6889 | 1.9862 |
| 1203.2321 | 0.5465 | 1201.3917 | 0.9631 | 1211.2323 | 0.8609 |
| 1240.8765 | 11.2817 | 1242.4834 | 9.7173 | 1249.6025 | 10.899 |
| 1273.9853 | 98.2027 | 1293.5511 | 80.1176 | 1294.2503 | 87.3926 |
| 1343.9336 | 57.7804 | 1357.207 | 6.9377 | 1366.703 | 98.4908 |
| 1355.9973 | 6.9596 | 1361.8617 | 101.873 | 1367.5971 | 7.8376 |
| 1418.4209 | 14.3997 | 1432.3272 | 17.2693 | 1439.9916 | 20.7159 |
| 1432.9714 | 0.7497 | 1443.6093 | 2.308 | 1452.6938 | 2.4449 |
| 1479.057 | 0.0034 | 1496.2766 | 0.0848 | 1500.4568 | 0.0036 |
| 1495.829 | 0.2058 | 1511.3708 | 1.1689 | 1514.7886 | 0.6065 |
| 1560.3264 | 4.4354 | 1588.4153 | 4.3186 | 1592.9353 | 4.3834 |
| 1602.2546 | 12.7333 | 1633.2842 | 11.3316 | 1636.6116 | 11.1114 |
| 3098.6556 | 30.7548 | 3144.2036 | 31.1503 | 3129.3535 | 25.5008 |
| 3104.2268 | 2.6267 | 3148.836 | 1.0869 | 3135.1695 | 3.1176 |
| 3123.2312 | 106.811 | 3168.6695 | 81.7649 | 3153.6619 | 107.7872 |
| 3136.1162 | 26.0687 | 3180.2098 | 27.0673 | 3161.4406 | 29.1727 |
| 3143.0253 | 92.101 | 3187.1713 | 68.4009 | 3173.0392 | 89.482 |
| 3166.3196 | 77.4721 | 3213.3687 | 56.8191 | 3193.0846 | 75.0111 |
| 3176.5322 | 43.0562 | 3222.4879 | 32.7072 | 3203.5548 | 43.1038 |

File S2.2: Unscaled harmonic frequencies of fluorenyl anion $C_{13}H_9^-$.

| B3LYP–D3 | | M062X–D3 | | wB97XD | |
|---|---|---|---|---|---|
| 108.6634 | 2.3779 | 109.2452 | 2.8283 | 110.0196 | 2.8527 |
| 142.3566 | 0.0 | 142.4674 | 0.0 | 143.6257 | 0.0 |
| 217.9963 | 2.0712 | 211.3186 | 2.0747 | 217.3184 | 2.1877 |
| 288.3734 | 0.0042 | 288.5127 | 0.0001 | 290.413 | 0.0041 |
| 295.9484 | 0.0 | 297.6488 | 0.0 | 299.4622 | 0.0 |
| 433.1293 | 2.0942 | 433.5105 | 1.9834 | 436.325 | 2.2745 |
| 435.2318 | 12.1068 | 436.7539 | 13.063 | 439.9801 | 13.2243 |
| 450.8939 | 0.0 | 452.0982 | 0.0 | 455.885 | 0.0 |
| 502.5383 | 10.1965 | 496.4552 | 11.5849 | 504.7823 | 12.5395 |
| 550.0087 | 0.1983 | 546.9782 | 0.5795 | 553.6853 | 0.5085 |
| 580.6253 | 16.9929 | 586.9937 | 16.33 | 590.9785 | 18.0841 |
| 593.7304 | 0.0 | 598.3372 | 0.0 | 603.0921 | 0.0 |
| 627.1911 | 1.2792 | 624.7458 | 1.5804 | 631.3004 | 1.1857 |
| 650.3686 | 0.0744 | 648.2121 | 0.0508 | 654.1515 | 0.0563 |
| 651.54 | 7.8138 | 669.1633 | 7.4474 | 654.9148 | 11.6867 |
| 720.4241 | 144.8881 | 731.7905 | 133.4867 | 733.1331 | 143.3615 |
| 725.0266 | 0.0 | 738.4904 | 0.0 | 738.9242 | 0.0 |
| 747.2392 | 0.1069 | 753.5829 | 0.0658 | 756.2958 | 0.1012 |
| 760.4725 | 19.0074 | 772.437 | 31.5484 | 773.2456 | 23.3721 |
| 779.6781 | 0.0 | 789.0995 | 0.0 | 792.3624 | 0.0 |
| 832.9921 | 7.2592 | 848.8842 | 0.0686 | 849.9563 | 8.2501 |
| 839.2695 | 0.0 | 848.9533 | 7.6805 | 853.7928 | 0.0033 |
| 843.2811 | 1.0174 | 853.7951 | 0.0 | 855.4618 | 0.0 |
| 884.5485 | 0.1287 | 886.1515 | 0.1146 | 892.9699 | 0.0635 |
| 924.2054 | 3.6095 | 944.9221 | 2.9501 | 945.5775 | 3.1952 |
| 928.7605 | 0.0 | 946.2714 | 0.0 | 949.0159 | 0.0 |
| 948.0575 | 0.0 | 969.1796 | 0.0 | 970.1235 | 0.0 |
| 949.615 | 0.0188 | 971.2297 | 0.053 | 971.6539 | 0.0162 |
| 999.6785 | 90.6014 | 1001.9982 | 65.9291 | 1008.2474 | 84.0434 |
| 1020.3491 | 7.8474 | 1029.0568 | 16.8482 | 1034.8915 | 8.2658 |
| 1022.0906 | 6.0644 | 1030.9488 | 7.5905 | 1035.0077 | 10.0015 |
| 1115.8978 | 0.6887 | 1118.3436 | 0.4149 | 1126.39 | 0.6102 |
| 1123.3481 | 36.0265 | 1127.6307 | 33.4201 | 1134.3876 | 36.9134 |
| 1154.8265 | 10.5062 | 1144.1783 | 9.0544 | 1157.2073 | 10.2349 |

| | | | | | |
|---|---|---|---|---|---|
| 1165.859 | 8.7929 | 1150.7125 | 8.1182 | 1164.4177 | 9.1234 |
| 1169.9649 | 0.2852 | 1158.8744 | 1.0049 | 1171.7332 | 0.5851 |
| 1222.18 | 10.6852 | 1220.9025 | 9.3034 | 1229.9283 | 10.7482 |
| 1244.8247 | 130.0378 | 1248.7828 | 133.1594 | 1257.2107 | 141.2732 |
| 1314.698 | 4.2256 | 1330.6552 | 0.0465 | 1336.2832 | 1.3388 |
| 1333.4258 | 2.6659 | 1344.1816 | 10.7942 | 1347.9544 | 10.5611 |
| 1346.4064 | 163.0208 | 1349.7707 | 234.446 | 1358.9176 | 229.3913 |
| 1363.1255 | 5.8514 | 1358.7755 | 5.1965 | 1367.3472 | 5.4851 |
| 1403.279 | 3.8606 | 1405.4371 | 2.9997 | 1414.6381 | 2.8853 |
| 1456.6643 | 17.5301 | 1473.0507 | 22.6746 | 1480.9791 | 26.9215 |
| 1467.8637 | 18.7878 | 1476.4189 | 29.8404 | 1485.2866 | 32.2222 |
| 1494.6491 | 0.0777 | 1510.9412 | 0.0006 | 1514.3274 | 0.0412 |
| 1500.8825 | 84.248 | 1511.7273 | 125.5531 | 1517.1606 | 126.4142 |
| 1560.5035 | 0.5987 | 1592.1417 | 0.1937 | 1596.3061 | 0.2448 |
| 1568.1435 | 0.6018 | 1592.1797 | 0.8117 | 1597.5973 | 1.2311 |
| 1596.9276 | 103.8975 | 1625.8144 | 107.7682 | 1629.5935 | 117.9815 |
| 1630.5058 | 27.9639 | 1663.9587 | 30.3398 | 1668.815 | 30.592 |
| 3110.8164 | 2.2557 | 3154.5075 | 2.2187 | 3140.7556 | 3.2184 |
| 3111.2859 | 17.4019 | 3155.0721 | 18.4171 | 3141.0951 | 15.8976 |
| 3117.8439 | 4.7802 | 3159.4134 | 4.766 | 3147.7892 | 6.6881 |
| 3121.4383 | 2.0764 | 3161.7461 | 6.0213 | 3149.7554 | 3.3731 |
| 3133.4327 | 55.0721 | 3176.3102 | 50.2444 | 3163.6104 | 49.174 |
| 3134.0697 | 140.2356 | 3176.7267 | 99.5329 | 3164.0427 | 140.6546 |
| 3153.3756 | 132.384 | 3196.5928 | 90.6987 | 3182.7283 | 132.1879 |
| 3154.5071 | 32.7155 | 3197.3503 | 25.5889 | 3183.7139 | 28.6892 |
| 3175.6663 | 37.9793 | 3221.4872 | 25.8399 | 3203.7317 | 34.7874 |

## S2.2 Anharmonic (indenyl cation)

File S2.3: Anharmonic frequencies of indenyl cation $C_9H_7^+$ (M06-2X-D3/aug-cc-pVTZ). Note: for full output consult the text file in the dataset.

Fundamental Bands

| Mode(n) | E(harm) | E(anharm) | I(harm) | I(anharm) |
|---|---|---|---|---|
| 1(1) | 3278.947 | 3186.814 | 19.27006826 | 4.34387605 |
| 2(1) | 3240.664 | 3165.100 | 2.63830035 | 0.45858055 |
| 3(1) | 3227.891 | 3227.980 | 0.53162324 | 4.23363955 |
| 4(1) | 3218.684 | 3163.834 | 0.00253209 | 0.98562171 |
| 5(1) | 1714.252 | 1654.201 | 84.31705997 | 39.15908468 |
| 6(1) | 1567.115 | 1523.674 | 118.01269361 | 25.84180855 |
| 7(1) | 1511.062 | 1474.661 | 0.67929549 | 1.82782668 |
| 8(1) | 1437.520 | 1407.533 | 4.43812270 | 1.26403038 |
| 9(1) | 1218.086 | 1147.494 | 132.23737855 | 40.11487595 |
| 10(1) | 1203.250 | 1210.632 | 1.14674663 | 13.36716870 |
| 11(1) | 1104.318 | 1109.812 | 33.81804031 | 56.21502177 |
| 12(1) | 1010.449 | 961.812 | 19.50288825 | 3.04100913 |
| 13(1) | 874.181 | 868.910 | 0.35205164 | 1.55881100 |
| 14(1) | 747.152 | 735.410 | 27.34501003 | 18.95742268 |
| 15(1) | 542.721 | 536.540 | 0.22454114 | 1.36946965 |
| 16(1) | 1045.794 | 1131.988 | 0.00000000 | 0.00000047 |
| 17(1) | 1032.859 | 1050.740 | 0.00000000 | 0.00003352 |
| 18(1) | 924.691 | 930.489 | 0.00000000 | 0.00000653 |
| 19(1) | 743.369 | 769.178 | 0.00000000 | 0.00003096 |
| 20(1) | 502.950 | 521.740 | 0.00000000 | 0.00002452 |
| 21(1) | 224.552 | 245.632 | 0.00000000 | 0.00001378 |
| 22(1) | 1027.760 | 1025.725 | 0.09111448 | 0.49006552 |
| 23(1) | 999.715 | 1012.775 | 3.06853000 | 0.12293511 |
| 24(1) | 825.992 | 784.340 | 51.43975111 | 3.44677304 |
| 25(1) | 772.721 | 807.663 | 27.21884216 | 73.85462154 |
| 26(1) | 433.481 | 489.612 | 22.82945546 | 15.94471628 |
| 27(1) | 356.482 | 372.043 | 9.78300911 | 15.52216893 |
| 28(1) | 171.819 | 165.075 | 5.77202506 | 5.82455053 |
| 29(1) | 3230.042 | 3213.905 | 8.93417565 | 5.16004031 |
| 30(1) | 3228.767 | 3176.911 | 1.61362853 | 146.19695407 |
| 31(1) | 3216.393 | 3165.572 | 1.08053363 | 114.90016819 |
| 32(1) | 1638.153 | 1578.787 | 5.50637562 | 5.23112684 |
| 33(1) | 1464.276 | 1452.711 | 63.89556330 | 51.65903792 |
| 34(1) | 1409.229 | 1356.192 | 119.66483995 | 78.29403116 |
| 35(1) | 1327.829 | 1300.142 | 35.91178555 | 42.78481919 |
| 36(1) | 1249.171 | 1232.070 | 40.66111262 | 1.86576740 |
| 37(1) | 1164.542 | 1133.758 | 7.99597040 | 8.17504128 |

| | | | | |
|---|---|---|---|---|
| 38(1) | 1075.854 | 1055.174 | 17.39505097 | 14.54013039 |
| 39(1) | 978.137 | 967.192 | 48.30000787 | 37.58308199 |
| 40(1) | 843.117 | 829.525 | 37.20868663 | 20.27663461 |
| 41(1) | 490.304 | 491.786 | 27.75112021 | 20.09884060 |
| 42(1) | 327.975 | 347.238 | 26.02206831 | 12.02930204 |

# S3 Band Assignments of IRPD Spectra

Table S3.1: Band positions of the experimental IRPD spectra, $\bar{\nu}_{ex}$, are compared to the theoretically computed fundamental transitions (harmonic calculations), $\bar{\nu}_{th}$, of the indenyl and fluorenyl anions and their bandshifts, $\Delta\bar{\nu}$, are noted.

| **IRPD Spectrum** | **M06-2X-D3/aug-cc-pVTZ** | | | | |
|---|---|---|---|---|---|
| **$C_9H_7^-$** | **Indenyl Anion** | | | | |
| $\bar{\nu}_{ex}$ [cm$^{-1}$] | $\bar{\nu}_{th}$ [cm$^{-1}$] | | $\Delta\bar{\nu}$ [cm$^{-1}$] | **Mode** | **Notes** |
| 658 | 663 | | -5 | $\gamma_{CH}$ | |
| 709 | 715 | | -6 | $\gamma_{CH}$ | |
| 854 | 843 | | 11 | $\nu_{CC}$ | |
| 992 | 993 | | -1 | $\nu_{CC}, \delta_{CH}$ | |
| 1015 | 1000 | | 15 | $\nu_{CC}, \delta_{CH}$ | Assignment unclear. |
| 1031 | 1025 | | 6 | $\nu_{CC}, \delta_{CH}$ | |
| 1207 | 1201 | | 6 | $\nu_{CC}, \delta_{CH}$ | |
| 1249 | 1251 | | -2 | $\nu_{CC}, \delta_{CH}$ | |
| 1290 | – | | – | | Missing in calc. |
| 1328 | 1317 | | 11 | $\nu_{CC}, \delta_{CH}$ | |
| 1386 | 1385 | | 1 | $\nu_{CC}, \delta_{CH}$ | |

| **$C_{13}H_9^-$** | **Fluorenyl Anion** | | | | |
|---|---|---|---|---|---|
| $\bar{\nu}_{ex}$ [cm$^{-1}$] | $\bar{\nu}_{th}$ [cm$^{-1}$] | | $\Delta\bar{\nu}$ [cm$^{-1}$] | **Mode** | **Notes** |
| 703 | 708 | | -5 | $\gamma_{CH}$ | |
| 735 | 747 | | -12 | $\gamma_{CH}$ | |
| 809 | 821 | | -12 | $\gamma_{CH}$ | |
| 979 | 969 | | 10 | $\nu_{CC}, \delta_{CH}$ | |
| 1000 | 995 | | 5 | $\nu_{CC}, \delta_{CH}$ | |
| 1096 | 1090 | | 6 | $\nu_{CC}, \delta_{CH}$ | |
| 1123 | 1113 | | 10 | $\nu_{CC}, \delta_{CH}$ | |
| 1216 | 1208 | | 8 | $\nu_{CC}, \delta_{CH}$ | |
| 1316 | 1305 | | 11 | $\nu_{CC}, \delta_{CH}$ | |
| 1421 | 1424 | (–) | -3 | $\nu_{CC}, \delta_{CH}$ | Assignment unclear. |
| 1461 | 1462 | (1424) | -1 | $\nu_{CC}, \delta_{CH}$ | Assignment unclear. |
| 1487 | – | (1462) | – | $\nu_{CC}, \delta_{CH}$ | Assignment unclear. |
| 1563 | 1572 | | -9 | $\nu_{CC}, \delta_{CH}$ | |
| 1605 | 1609 | | -4 | $\nu_{CC}, \delta_{CH}$ | |

**Note:** Modes are described as follows: $\gamma_{CH}$ – out-of-plane CH bending mode, $\delta_{CH}$ – in-plane CH bending mode, and $\nu_{CC}$ – CC stretching mode. Calculated frequencies were scaled by 0.967.

# S4 Nucleus-Independent Chemical Shifts (NICS)

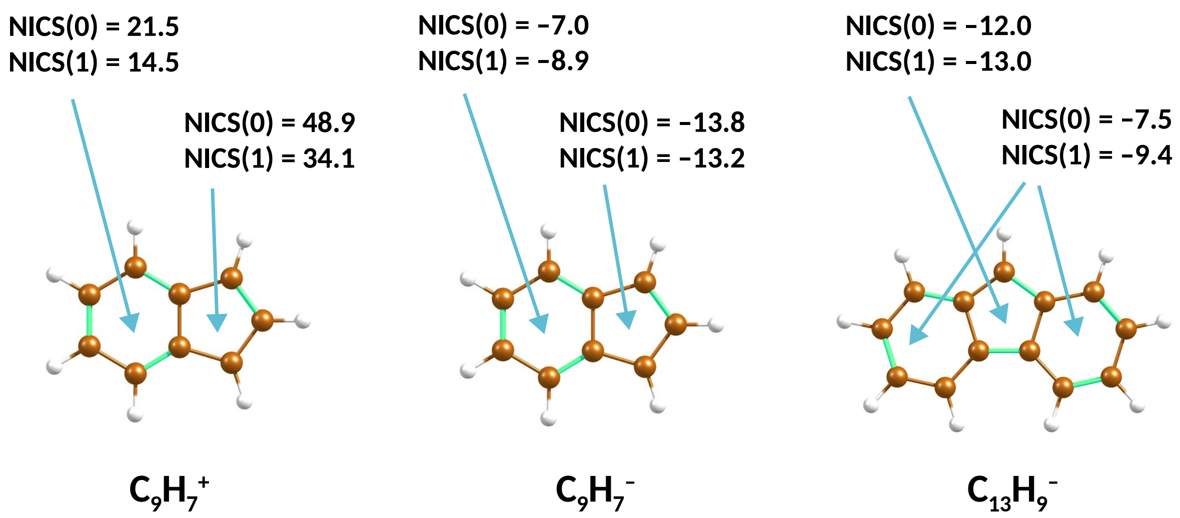


Figure S4.1: NICS values given in ppm (unit) as calculated at the M06-2X/aug-cc-pVTZ level. In parenthesis, the distance from the molecular plane is given.

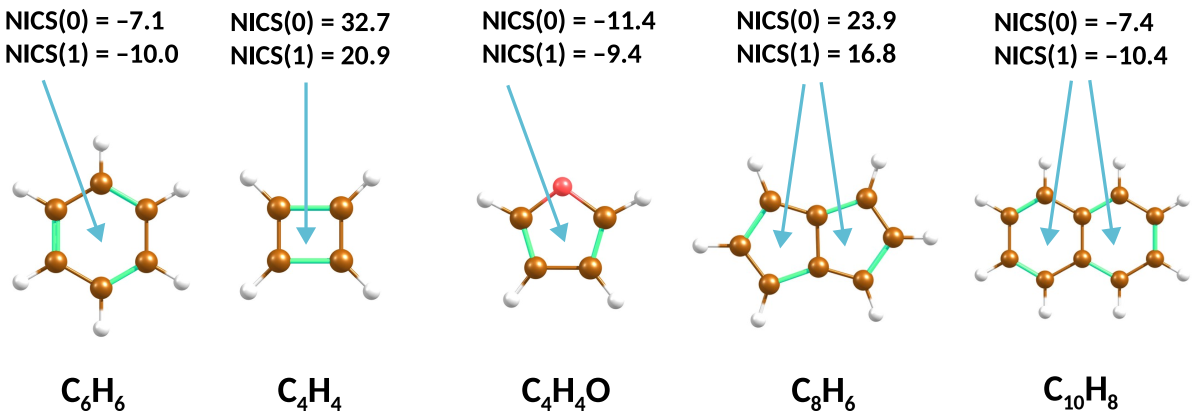


Figure S4.2: NICS values in ppm in other species for comparison as calculated at the M06-2X/aug-cc-pVTZ level. In parenthesis, the distance from the molecular plane is given.

# S5 Possibility of Isomers

## S5.1 Indenyl Anion

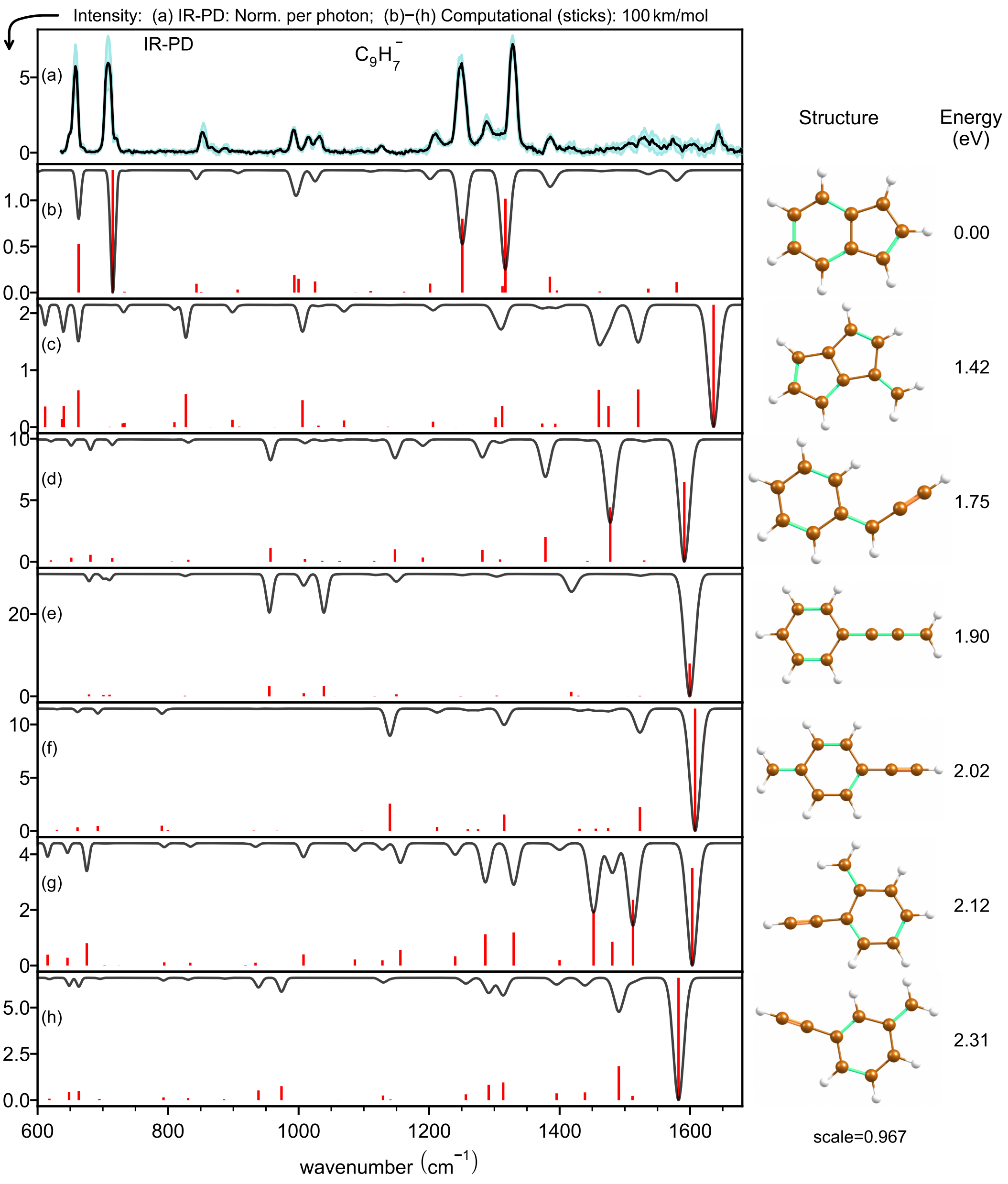


Figure S5.1: Structures, relative stability, and vibrational spectra of various $C_9H_7^-$ anions as calculated at the M06-2X-D3/aug-cc-pVTZ level.

## S5.2 Indenyl Cation

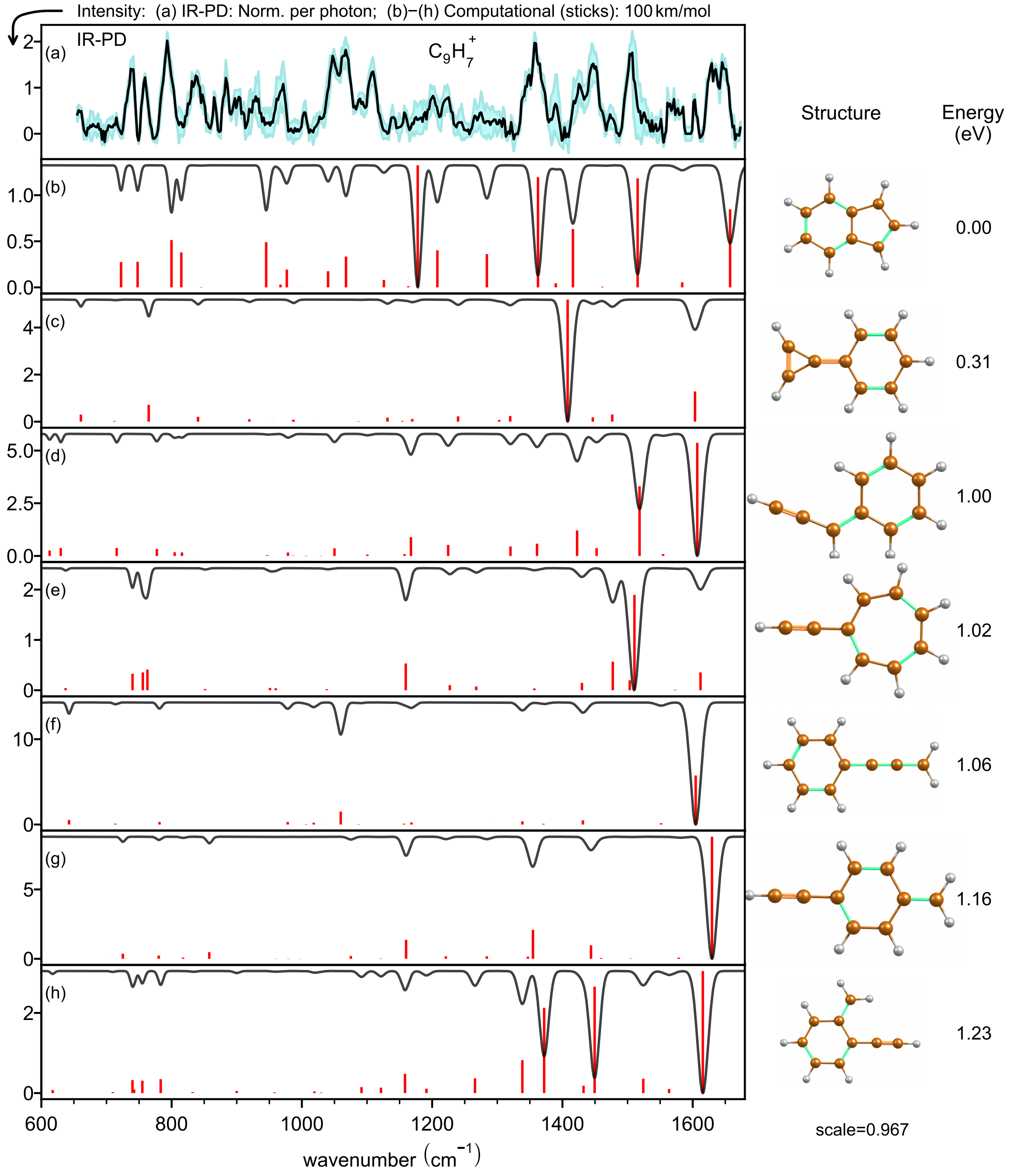


Figure S5.2: Structures, relative stability, and vibrational spectra of various $C_9H_7^+$ ions as calculated at the M06-2X-D3/aug-cc-pVTZ level.
