# Supplementary material for: Infrared Spectroscopy of Pentagon-Containing PAHs: Indenyl and Fluorenyl Anions and Indenyl Cation
Source: J Phys Chem Lett. 2025 Apr 11;16(16):3938–44. doi: 10.1021/acs.jpclett.5c00570 (PMC12035856; doi:10.1021/acs.jpclett.5c00570)
Supplement: Supplementary file 2 — jz5c00570_si_002.pdf [file jz5c00570_si_002.pdf]

jz-2025-00570m.R1

Name: Peer Review Information for "Infrared Spectroscopy of Pentagon-Containing PAHs: Indenyl and Fluorenyl Anions and Indenyl Cation"

First Round of Reviewer Comments

Reviewer: 1

Comments to the Author

I would like to thank the editor for assigning this paper to me, and restoring my belief in the outstanding research going on in the gas-phase community. This manuscript describing the vibrational predissociation spectra of these astrochemically relevant ions was superbly written. I recommend accepting as is.

Reviewer: 2

Comments to the Author

This manuscript presents a detailed spectroscopic investigation of pentagon ring containing polycyclic aromatic hydrocarbon (PAH) ions, focusing on the indenyl ($C_9H_7^-$) and fluorenyl ($C_{13}H_9^-$) anions as well as the indenyl cation ($C_9H_7^+$). Using infrared pre-dissociation (IRPD) spectroscopy in a cryogenic ion trap coupled with a free-electron laser at the FELIX Laboratory, the authors obtained the first experimental gas-phase infrared spectra of these astrochemically relevant species.

The study combines these experimental results with density functional theory (DFT) calculations and molecular dynamics simulations to interpret the observed vibrational features. The spectra of the anionic PAHs were successfully assigned through comparison with DFT calculations, revealing strong vibrational bands near 1300 cm^{-1} (7.7 μm), which corresponds to a prominent emission feature observed in many astronomical objects.

The work carries significant implications for astrochemistry, particularly in understanding the spectroscopic signatures of PAHs in space. The experimental data provided here serve as valuable benchmarks for interpreting astronomical observations, especially with the advent of high-resolution telescopes like JWST.

This paper is recommended for publication with minor revisions:

1. Regarding the unassigned peaks of the indenyl anion: Have the authors looked at predicted spectra possible isomers, as they did for the cation? Given that the DFT calculations align well with the experimental spectra of the anions, identifying an isomer whose calculated spectrum matches the unassigned features could rule out the possibility of these peaks arising from combination bands or anharmonic shifts.

2. Line 122,183: The citation appears before the period.

Author's Response to Peer Review Comments:

Max-Planck-Institut fur extraterrestrische Physik ̈

The Journal of Physical Chemistry Letters
Editorial Office

Garching b. Munchen, April 3, 2025 ̈

Dear Editor

Thank you for considering our manuscript entitled *“Infrared Spectroscopy of PentagonContaining PAHs: Indenyl and Fluorenyl Anions and Indenyl Cation”* for publication in The Journal of Physical Chemistry Letters.

We thank the two reviewers for their reviews.

Please find the answers to reviewers’ comments attached (next page). The comments/ questions are typeset in roman letters, while our answers are typset in *italics*.

On top of these, we have only made a minor correction around one reference to previous works, which has been omitted, by mistake, during the final preparation of the 1. submission. Sincerely,

Pavol Jusko

Zentrum fur¨ astrochemische Studien (CAS)

Dr. Pavol Jusko
Gießenbachstraße 1
85748 Garching Germany

tel. +49 89 30000-3386 fax. +49 89 30000-3569 e. pjusko@mpe.mpg.de

Reviewer 1.:

Recommendation: This paper represents a significant new contribution and should be published as is.

Comments: I would like to thank the editor for assigning this paper to me, and restoring my belief in the outstanding research going on in the gas-phase community. This manuscript describing the vibrational predissociation spectra of these astrochemically relevant ions was superbly written. I recommend accepting as is.

We thank reviewer 1 for their very positive assessment.

Reviewer 2.:

Recommendation: This paper is publishable subject to minor revisions noted. Further review is not needed.

Comments: This manuscript presents a detailed spectroscopic investigation of pentagon ring containing polycyclic aromatic hydrocarbon (PAH) ions, focusing on the indenyl ($C_9H_7^-$) and fluorenyl ($C_{13}H_9^-$) anions as well as the indenyl cation ($C_9H_7^+$). Using infrared pre-dissociation (IRPD) spectroscopy in a cryogenic ion trap coupled with a free-electron laser at the FELIX Laboratory, the authors obtained the first experimental gas-phase infrared spectra of these astrochemically relevant species. The study combines these experimental results with density functional theory (DFT) calculations and molecular dynamics simulations to interpret the observed vibrational features. The spectra of the anionic PAHs were successfully assigned through comparison with DFT calculations, revealing strong vibrational bands near 1300cm^{-1} (7.7μm), which corresponds to a prominent emission feature observed in many astronomical objects. The work carries significant implications for astrochemistry, particularly in understanding the spectroscopic signatures of PAHs in space. The experimental data provided here serve as valuable benchmarks for interpreting astronomical observations, especially with the advent of high-resolution telescopes like JWST.

We thank the reviewer for their careful reading and suggestions.

This paper is recommended for publication with minor revisions:

1. Regarding the unassigned peaks of the indenyl anion: Have the authors looked at predicted spectra possible isomers, as they did for the cation? Given that the DFT calculations align well with the experimental spectra of the anions, identifying an isomer whose calculated spectrum matches the unassigned features could rule

out the possibility of these peaks arising from combination bands or anharmonic shifts.

This is a very good point. We have added a figure S5.1 in the SI, that is analogous to the figure S5.2 (isomers of indenyl cation), for 7 isomers of $C_9H_7^-$. The next closest isomer is >1 eV higher in energy and the calculated spectra show no hint that this could be the case. Therefore, we modified/changed the affected sentence in the manuscript to:

Although we cannot completely rule out the presence of another $C_9H_7^-$ isomer in low abundance/concentration, none of the calculated spectra of selected isomers indicate that this would be the case (see Fig. S5.1 in the SI).

2. Line 122,183: The citation appears before the period. *Fixed.*
